# The Long-Term Effect of Blue-Light Blocking Spectacle Lenses on Adults’ Contrast Perception

**DOI:** 10.3389/fnins.2022.898489

**Published:** 2022-07-15

**Authors:** Yan Lian, Weiwei Lu, Haixiao Huang, Ge Wu, Aiqin Xu, Wanqing Jin

**Affiliations:** State Key Laboratory of Ophthalmology, Optometry and Vision Science, School of Ophthalmology and Optometry, Affiliated Eye Hospital, Wenzhou Medical University, Wenzhou, China

**Keywords:** contrast perception, long-term effect, blue-light blocking, spectacle, lenses

## Abstract

**Purpose:**

To evaluate the long-term effect of two different degrees of blue-light blocking (BB) spectacle lenses on adults’ contrast perception under various lighting conditions.

**Methods:**

In total, 144 healthy adults aged 24.70 (±4.32 years) were recruited to this randomized controlled trial. The participants were randomly divided into three groups and used three different spectacle lenses (15% BB: 15% blue-blocking spectacle lenses; 30% BB: 30% blue-blocking spectacle lenses; RC: regular clear lenses serving as control). Contrast sensitivity under four light conditions (scotopic and photopic, both with/without glare) was measured using standard clinical tests at baseline, 1 month, 3 months and 6 months of use. The area under the log contrast sensitivity function (AULCSF) was also computed as an index for their overall contrast sensitivity across spatial frequencies.

**Results:**

There was no significant difference in AULCSFs among the three types of spectacle lenses under any light condition (all *P* > 0.81). No statistical difference was found in the AULSCF among the four time points (all *P* > 0.39), with no interaction between the effects of group and time (all *P* > 0.42).

**Conclusion:**

Wearing blue-light blocking lens had no clinically significant effect on adults’ long-term contrast perception under scotopic or photopic conditions, or with glare.

## Introduction

Blue light is a visible light with a short wavelength ranging from approximately 380 to 500 nm. It highly penetrative due to its high energy. Blue light is emitted by common light-emitting diodes (LED) and common electronic devices. Long-term exposure to high-energy blue light may damage the eye and circadian rhythm. *In vitro* and *in vivo* studies indicate that blue light can induce oxidative damage and apoptosis in retinal pigment epithelial (RPE) cells, thereby leading to age-related macular degeneration (AMD) (Kaarniranta et al., 2018; Blasiak, 2020; Luo et al., 2021). Blue light has also been shown to induce eye fatigue (Ide et al., 2015; Singh et al., 2021) and disrupt the circadian rhythm in humans (Chellappa et al., 2011). Due to its potentially deleterious effect, it has caught the attention of the research community to investigate more about its effect on the vision and physiology of human adults and possible means to filter out the blue-light (i.e., blue-light filtration).

The most common technology used in blue-light filtration is the blue light-blocking (BB) spectacle lens. In theory, BB lenses provide the retinal protection against photochemical damage. Its ability for macular protection has been corroborated in several animal and cell studies (Alzahrani et al., 2020; Blasiak, 2020; Luo et al., 2021; Sanchez-Ramos et al., 2021). However, this theory has only been weakly supported in the human literature (Lawrenson et al., 2017). To illustrate, a large cohort study (Achiron et al., 2021) with 11397 eyes shows that the blue light–filtering intraocular lenses (IOL) has no apparent advantage in the incidence and progression of AMD. However, there have been some previous clinical studies that demonstrate that BB spectacle lenses can reduce the symptoms of eye strain in individuals who use digital devices (Ide et al., 2015; Lin et al., 2017). BB lenses may also improve sleep quality if the user wears them at night (Lawrenson et al., 2017; Esaki et al., 2020).

However, there has been increasing anxiety regarding the potential influence of technology of blue-light filtration on visual perception. Leung et al. (2017) measured the contrast sensitivity after the observers wore a new pair of BB spectacle lenses and found similar visual perception to those wore clear lenses. Their results reveal that wearing BB lenses for a short period of time does not change observer’s (intraday) contrast sensitivity. It is possible that the effect of BB lenses does not show immediately as the human visual system may require more time to adapt to the changes. For example, Gao et al. (2018) found an improvement in visual function after 18-weeks of refractive adaptation. In addition, Chen et al. (2007) recorded the normalization of visual acuity after 6 months of optical adaptation in amblyopia. Therefore, an important issue that remains unaddressed is whether BB spectacle lenses affect long-term visual perception. In particular, do these BB spectacle lenses reduce or improve scotopic or photopic contrast sensitivity, with or without glare? To address this question, we examined the long-term effect of BB spectacle lenses on adults’ contrast sensitivity (CS) under multiple light environments and compared it with the long-term effect of wearing regular clear spectacle lenses.

## Materials and Methods

### Optical Parameters of Three Types of Lenses

Two common types of blue-light blocking (BB) spectacle lenses (refractive index = 1.56) were evaluated (one was “Qin Zhiyu,” Wanming Optical Co., Ltd., China; the other one was “Meijing,” Mingyue Optical Co., Ltd., China). A regular clear lens (refractive index = 1.56) served as a control lens (“Baolijing,” Wanming Optical Co., Ltd., China). Their spectral transmittances, T (λ), within the wavelength (λ) range of 280 to 780 nm, were measured (measured step size = 1 nm; calculated step size = 5 nm) by a UV/V spectrophotometer (HITACHI-U4100, China). The reflectivity, absorptivity, and yellow index of three kinds of spectacle lenses were also measured to identify how the BB lenses filtered blue light (reflecting blue light or absorbing blue light). Details are presented in Table 1. In this paper, we refer the two BB lenses as “15% BB” and “30% BB” and the control lenses as RC (regular clear lenses).

**TABLE 1:** Optical detection parameters of three types of lenses.

Types of lenses	RC	15% BB	30% BB
UV-transmittance (%) (315–380 mm)	0.20	0.10	0.00
Blue light transmittance (%) (380–500 nm)	97.70	84.40	69.60
Other visible spectral transmittance (%) (500–780 mm)	97.99	98.42	92.10
Reflectivity (%)	2.51	4.43	3.90
Absorptivity (%)	5.95	8.98	12.38
Yellow index (YI)	–0.07	9.41	31.32

*RC: regular clear lenses serving as a control; 15% BB: 15% blue-blocking spectacle lenses; 30% BB: 30% blue-blocking spectacle lenses.*

### Essential Information

A randomized controlled clinical trial was conducted to evaluate the long-term effect in visual performance of those who wore the spectacles. This study was registered with the Ethics Committee of the Eye Hospital of Wenzhou Medical University for clinical trials (KYK [2015]13). The study design met the WHO’s definition of a clinical trial, as stipulated in the Declaration of Helsinki. The clinical registration code was ChiCTR1800020191.

A total of 150 healthy adults who wore spectacles habitually volunteered to participate in the experiment. They were followed up for 6 months (a total of four visits). The participants provided an informed consent. The inclusion criteria were: (1) age between 18 and 30 years; (2) spherical refraction < −6.00 D and astigmatism < −1.5 D; (3) monocular corrected distance visual acuity (logMAR) equal to or less than 0.0; (4) experience of wearing spectacles for more than 6 h a day. The exclusion criteria were: (1) eye diseases except for refractive errors, especially retinal or macular diseases; (2) experience of corneal contact lens wearing within 1 month; (3) ocular surgery or disease; (4) psychological or systemic diseases. The participants were divided into three groups by generating random numbers, and each group was randomly assigned one type of spectacle lens. They wore their assigned lenses for 6 months. Contrast sensitivity was performed at the initial visit and at 1, 3, and 6 months.

### Contrast Sensitivity

Contrast sensitivity was measured using the CSV-1000 contrast sensitivity test (VectorVision, Ohio, United States) at 2.5 m with the spectacle lenses. Contrast sensitivity was evaluated at 3, 6, 12, and 18 cycles per degree (cpd) under four light conditions: scotopic (3 cd/cm^2^) and photopic (85 cd/cm^2^), both with/without glare (which was set as 80% of glare). The order of measurement of the four kinds of light conditions was fixed and a 10-min rest period was implemented to allow for adaptation to the new light condition. The area under the log contrast sensitivity function (AULCSF) computed by fitting the contrast sensitivity function with the given data based on the method outlined by Applegate et al. (1998). We plotted the log of CS as a function of log spatial frequency and fitted third-order polynomials to the data. The fitted function between the fixed limits of log spatial frequencies at 3 cycles/degree to log spatial frequencies at 18 cycles/degree was used to compute the AULCSF.

### Statistical Analyses

All statistical analyses were performed using the SPSS software (ver. 25.0; SPSS Inc.). The measured data were tested using the Kolmogorov–Smirnov test. Data with a normal distribution are presented as the mean ± standard deviation (SD) in this report. The chi-square test was used to analyze the sex percentage between the three groups. Only the data of the right eye were selected for statistical analysis in this study because there was a high correlation between the two eyes in all parameters (all *P* < 0.05). The differences in baseline and demographic information among the three groups were analyzed using a one-way ANOVA. The two repeated-measures analysis of variance (RM-ANOVA) was used to compare the parameters of contrast sensitivity among the three groups with four different follow-up times. The level of statistical significance was set at *P* < 0.05.

## Results

### Baseline Data and Optical Parameter

A total of 150 participants were recruited; six withdrew from the experiment due to non-ophthalmic or spectacle-related reasons during the follow-up period, such as pregnancy and relocation. Therefore, 144 participants completed all follow-up visits, with a mean age of 24.7 ± 4.32 years (range, 20–39 years), including 36 males and 108 females. The mean spherical refraction was −3.35 ± 1.41 D (range, −0.50 D to −6.00 D) and a mean cylindrical refraction was −0.48 ± 0.48 D (range, 0.00 D to −1.50 D). No significant difference was found among the three groups in terms of demographic information and baseline ocular data (all *P* > 0.05; Table 2). No significant difference in visual acuity was found between baseline and 6 months of wearing the spectacle lenses (all *P* > 0.05). The optical parameters of the three lens types are listed in Table 2.

**TABLE 2:** Demographic information and baseline data of participants in three groups.

Groups	N	Gender, male [n(%)]	Age(Y)	BCVA (logMAR)	Equivalent refractive (D)	IOP (mmHg)
RC	45	11 (24.4%)	24.44 ± 4.55	−0.05 ± 0.06	−3.62 ± 1.49	14.28 ± 2.76
15% BB	49	11 (22.4%)	24.96 ± 4.27	−0.07 ± 0.08	−3.40 ± 1.56	15.14 ± 3.04
30% BB	50	14 (28.0%)	24.54 ± 3.77	−0.08 ± 0.06	−3.62 ± 1.28	14.58 ± 3.04
F/χ^2^	–	0.417	0.204	2.349	0.371	0.803
P	–	0.812	0.815	0.100	0.691	0.451

*BCVA: best corrected visual acuity; RC: regular clear lenses serving as a control; 15% BB: 15% blue-blocking spectacle lenses; 30%BB: 30% blue-blocking spectacle lenses.*

### Mean Contrast Sensitivities at Various Spatial Frequencies

**FIGURE 1:**
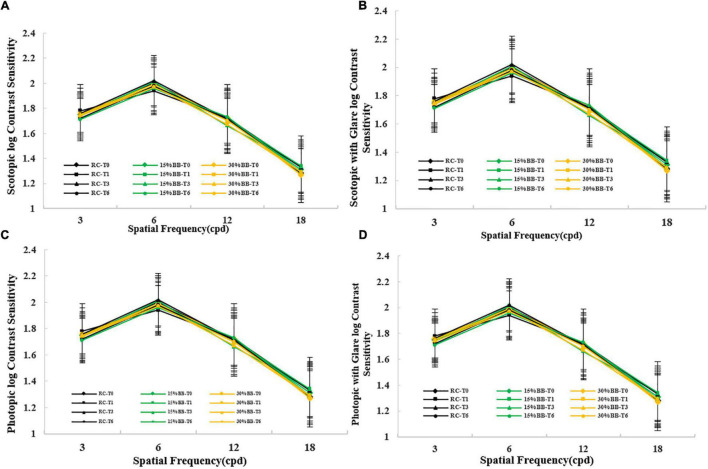
Contrast sensitivity under four kinds of condition in various spatial frequencies at four time points for three types of spectacle lenses. Error bars represent standard errors. **(A)** Contrast sensitivity under scotopic condition; **(B)** Contrast sensitivity under scotopic condition with glare; **(C)** Contrast sensitivity under photopic condition; **(D)** Contrast sensitivity under photopic condition with glare. RC: regular clear lenses; 15% BB:15% blue-blocking spectacle lenses; 30% BB: 30% blue-blocking spectacle lenses. T0 = baseline; T1 = 1 month; T3 = 3 months; T6 = 6 months.

Figure 1 shows the mean monocular contrast sensitivities from the three types of spectacle lenses across four spatial frequencies (3, 6, 12, and 18 cpd) under the four light conditions (scotopic, scotopic with glare, photopic, and photopic with glare) after 6-month of follow up visits. Repeated-measures ANOVA (Figure 1A) showed no significant difference in contrast sensitivity between the spectacle lenses in scotopic conditions at any spatial frequency (*P* = 0.831). There was no significant difference in scotopic contrast sensitivity between the time sessions (i.e., baseline, 1-month, 3-month, and 6-month visits) at any spatial frequency (*P* = 0.485). There was also no interaction among the effects of group, time and spatial frequencies (*P* = 0.921). Similarly, we also found no statistically significant difference (between the three kinds of spectacle lenses, and between the four follow-up visit times, or interaction among groups, times, and spatial frequencies) at any spatial frequency in the photopic conditions, scotopic with glare condition, and photopic with the glare condition (all *P* > 0.05; Figures 1B–D).

### Overall Area Under the Log Contrast Sensitivity Function

The area under the log contrast sensitivity function (AULCSF) of the three types of spectacle lenses, under four different light conditions, is shown in Table 3. During the 6 months, there was no significant difference among the three spectacle lenses in AULCSF, under any light condition (scotopic: *P* = 0.811; scotopic with glare: *P* = 0.908; photopic: *P* = 0.891; photopic with glare: *P* = 0.856). There was also no statistically significant difference in AULSCF among the four time sessions (scotopic: *P* = 0.754; scotopic with glare: *P* = 0.392; photopic: *P* = 0.433; photopic with glare: *P* = 0.564), with no interaction between the effects of group and time (scotopic: *P* = 0.428; scotopic with glare: *P* = 0.885; photopic: *P* = 0.829; photopic with glare: *P* = 0.576).

**TABLE 3:** Comparison of AULCSF of three types of spectacle lenses under different light conditions at four follow-up visits.

Visits	Scotopic			Scotopic + glare			Photopic			Photopic + glare		
	RC	15%BB	30%BB	RC	15%BB	30%BB	RC	15%BB	30%BB	RC	15%BB	30%BB
Baseline	1.43 ± 0.14	1.43 ± 0.12	1.41 ± 0.10	1.42 ± 0.21	1.41 ± 0.11	1.42 ± 0.10	1.46 ± 0.11	1.46 ± 0.10	1.45 ± 0.12	1.44 ± 0.10	1.44 ± 0.12	1.46 ± 0.11
1 month	1.42 ± 0.12	1.43 ± 0.12	1.41 ± 0.13	1.41 ± 0.13	1.41 ± 0.12	1.40 ± 0.12	1.47 ± 0.11	1.46 ± 0.12	1.46 ± 0.11	1.44 ± 0.10	1.46 ± 0.12	1.45 ± 0.10
3 months	1.43 ± 0.12	1.43 ± 0.13	1.42 ± 0.12	1.43 ± 0.12	1.41 ± 0.14	1.41 ± 0.13	1.44 ± 0.11	1.46 ± 0.11	1.45 ± 0.13	1.45 ± 0.10	1.46 ± 0.10	1.45 ± 0.11
6 months	1.42 ± 0.13	1.4 ± 0.13	1.42 ± 0.12	1.41 ± 0.12	1.40 ± 0.12	1.40 ± 0.13	1.45 ± 0.12	1.47 ± 0.10	1.46 ± 0.10	1.44 ± 0.10	1.44 ± 0.11	1.44 ± 0.09
*F*_*Inter–group*_	0.21			0.096			0.116			0.156		
P	0.811			0.908			0.891			0.856		
*F*_*Inter–time*_	0.571			1.002			0.916			0.682		
*P*	0.754			0.392			0.433			0.564		
*F*_*Group*×*Time*_	0.927			0.39			0.473			0.793		
*P*	0.428			0.885			0.829			0.576		

*It was analyzed with the repeated-measure ANOVA. Data are presented as the mean ± SD. RC group is the regular clear spectacle lenses; 15%BB group is 15% blue-light blocking spectacle lenses; 30%BB group is 30% blue-light blocking spectacle lenses.*

## Discussion

In this study, the long-term effect on visual perception of adults who wore BB lenses was assessed. The contrast sensitivity function with three kinds of spectacle lenses (RC, 15% BB, and 30% BB) under four types of light conditions (scotopic, photopic, scotopic with glare, photopic with glare) was measured at four time points (baseline, at 1-month, 3-month, and 6-month of spectacles use). Our results indicate that two different degrees of BB spectacles (15% blue filtering and 30% blue filtering) exhibited a similar degree of contrast sensitivity to that of the regular clear spectacles in adults. These results suggest that these two BB spectacle lenses showed no clinically detectable effect on the visual perception of adults in both short and long terms.

Previous studies on the effect of blue light filtering lenses on visual perception in adults have mainly focused on IOL or yellow filters. For example, Popov et al. (2021) and Hammond et al. (2019) both found that blue-light filtering IOLs did not have a clear influence on contrast sensitivity in elderly individuals. Lavric and Pompe (2014) also reported that blue-light filtering IOL did not affect the long-term contrast sensitivity of older observers for up to a year. In addition, Wang et al. (2010) reported that mesopic and photopic contrast sensitivities of adults were not affected by the yellow filter after refractive surgery. Mahjoob et al. (2015) also reported that the contrast sensitivity of adults under glare conditions was not altered by yellow filters. These studies indicate that the effect of blue light filtering on mesopic and photopic visual perception is minimal.

As for scotopic contrast sensitivity, Alzahrani et al. (2020) found that BB spectacle lenses reduced scotopic sensitivity by 5–24%. Indeed, some studies found a reduction in scotopic contrast sensitivity in older adults (Greenstein et al., 2007; Pierre et al., 2007). Blue light has a short wavelength (380–500 nm) that is close to the absorption peak of scotopic vision (506 nm) (Thapan et al., 2001). The reduced level of blue light that eventually reach the rod cell could be more likely to decrease the scotopic contrast sensitivity. In addition, some studies also report that blue light–filtering IOL could improve driving performance and reduce glare disability in older people under simulated glare conditions (Davison et al., 2011; Gray et al., 2011; Zhu et al., 2012).

One possibility for the contradictory results in these studies is the difference in age of the participants. Quentin et al. (2014) and Panda-Jonas et al. (1995) found that the reduction in contrast sensitivity by yellow filter was stronger in elderly individuals than in young individuals. Mahjoob et al. (2015) also showed that wearing yellow filter lenses improved visual acuity and contrast sensitivity under glare conditions. However, this was only found in older observers (aged 51–60 years old), not in young adults. Evidence suggests that rod cells decline with age (Panda-Jonas et al., 1995). This might explain why the influence of scotopic vision (related to rod cells by blue light filtration) is especially large in older adults. Nevertheless, our results, along with those of these previous reports, indicate that BB lenses are safe for young adults.

Although CSV-1000 contrast sensitivity test has been a standard tool to measure the contrast sensitivity in the clinic, it is unfortunately not sensitive to detect its minute changes. However, we believe that this is not a major issue of our study design as we believe that a too small change that is not detected by CSV-100 can be considered as clinically insignificant. Nevertheless, future studies should test the effect of the spectacles with a more sensitive tool that measures contrast sensitivity.

## Data Availability Statement

The raw data supporting the conclusions of this article will be made available by the authors, without undue reservation.

## Ethics Statement

The studies involving human participants were reviewed and approved by the Ethics Committee of Affiliated Eye Hospital, Wenzhou Medical University (2020-J-45). The patients/participants provided their written informed consent to participate in this study.

## Author Contributions

YL, WL, HH, GW, and WJ conceived the experiments. YL, WL, AX, and GW performed the experiments. YL, WL, and HH analyzed the data and interpreted the data. YL, WL, and WJ wrote the manuscript. All authors contributed to manuscript revision, read and approved the submitted version.

## Conflict of Interest

The authors declare that the research was conducted in the absence of any commercial or financial relationships that could be construed as a potential conflict of interest.

## Publisher’s Note

All claims expressed in this article are solely those of the authors and do not necessarily represent those of their affiliated organizations, or those of the publisher, the editors and the reviewers. Any product that may be evaluated in this article, or claim that may be made by its manufacturer, is not guaranteed or endorsed by the publisher.
